# “Anti-Bios”: Can Local Antibiotics Affect Bone Union in Infected Bone Defects Treated with Degradable Bone Substitutes

**DOI:** 10.3390/biomedicines13051070

**Published:** 2025-04-28

**Authors:** Filippo Vandenbulcke, Salvatore Lorenzo Renne, Giuseppe Anzillotti, Pietro Conte, Giuliano Ravasio, Gabriele Meroni, Federica Riva, Elizaveta Kon

**Affiliations:** 1 Department of Biomedical Sciences, Humanitas University, Via Rita Levi Montalcini 4, Pieve Emanuele, 20072 Milan, Italydoc.anzillotti@gmail.com (G.A.); pietro.conte@humanitas.it (P.C.);; 2 IRCCS Humanitas Research Hospital, Via Manzoni 56, Rozzano, 20089 Milan, Italy; 3 Department of Veterinary Medicine, University of Milan, Via Festa del Perdono 7, 20122 Milan, Italy; 4 Veterinary Teaching Hospital, University of Milan, Via dell’Università 6, 26900 Lodi, Italy; 5 Department of Biomedical, Surgical and Dental Sciences, University of Milan, Via Pascal 36, 20133 Milano, Italy; gabriele.meroni@unimi.it; 6 Department of Veterinary Medicine and Animal Science, University of Milan, Via dell’Universita 6, 26900 Lodi, ltaly; federica.riva@unimi.it

**Keywords:** infected bone defect, local antibiotics, fracture-related infections, bone healing, fracture repair

## Abstract

**Background**: Segmental bone defects (SBDs) pose significant clinical challenges, often requiring complex reconstructive procedures. Degradable bone substitutes loaded with antibiotics have emerged as promising tools for infection control. However, their impact on bone healing remains uncertain. This study investigates antibiotic-loaded biodegradable scaffolds in infected defects using an in vivo rabbit model. **Methods**: Thirty New Zealand white rabbits were divided into three groups—antibiotic-loaded GreenBone scaffolds, non-loaded GreenBone scaffolds, and allografts. A critical-size femoral defect was surgically created and inoculated with *Staphylococcus epidermidis*. Radiographic evaluations were performed over 16 weeks, followed by histological and microbiological analyses. Bone union, infection rates, and callus maturation were assessed. **Results**: Eight rabbits were excluded for technical errors. Bone union was significantly lower in the antibiotic-loaded group (two rabbits out of seven; 28.6%) compared to the non-loaded scaffold (13 rabbits out of 15; 86.7%; *p* = 0.006). The antibiotic-loaded group exhibited a higher incidence of chronic osteomyelitis (100%) versus non-loaded implants (60%; *p* < 0.05). Histological evaluation revealed delayed bone maturation in the antibiotic-loaded group (22.2% HOES grade 3) compared to non-loaded scaffolds (69.5%; *p* < 0.001). **Conclusions**: Despite their infection-fighting potential, antibiotic-loaded biodegradable scaffolds may impair bone healing, leading to higher non-union rates and delayed maturation. These findings highlight a critical trade-off between local antibiotic therapy and bone regeneration, warranting careful clinical consideration and further research to optimize treatment strategies for infected bone defects.

## 1. Introduction

Segmental bone defects (SBDs) pose significant challenges in orthopedic and reconstructive surgery and they are often defined as non-healing bone voids. SBDs often result from congenital malformations, high-energy trauma, tumour resections, infections, or complications arising from the treatment of complex non-unions [1]. Their treatment is critical not only for patient mobility and quality of life but also for alleviating the significant burden on healthcare systems. Indeed, recent data see bone grafting procedures accounting for over 10% of skeletal reconstructive surgeries globally, with an annual volume of 2–3 million procedures worldwide [2,3].

Traditional treatment modalities include autologous bone grafts, allografts, distraction osteogenesis, and synthetic bone substitutes [4,5]. Autologous grafts remain the gold standard due to their osteoinductive, osteoconductive, and osteogenic properties. However, their use is limited by donor-site morbidity, limited supply, and variable outcomes in infection-prone cases [3]. Allografts, while providing structural support and avoiding donor-site complications, carry risks of immune rejection, infection, and delayed incorporation [6]. Synthetic bone substitutes, such as calcium phosphate or bioactive glass-based materials, have emerged as promising alternatives due to their tunable properties and potential to act as drug delivery vehicles [7,8,9,10].

Among these, the GreenBone scaffold (GreenBone Ortho S. p.A.) has been proposed as a new synthetic solution composed of biomimetic substituted calcium phosphate phases (hydroxyapatite 85%+ β-tricalciumphosphate 15% and ions) with chemical and mechanical characteristics resembling those of native bone.

Nonetheless, infection still remains a major obstacle in the management of segmental bony defects. Loading bone substitutes with antibiotics could be an effective solution to reduce infectious risks but the effects of local antibiotics on bone regeneration are still not completely clarified [11]. Thus, the aim of this in vivo animal study on rabbit models is to investigate the effects of combining antibiotics with biodegradable bone substitutes in the management of infected bone defects. 

## 2. Materials and Methods

### 2.1. Study Design

**Figure 1:**
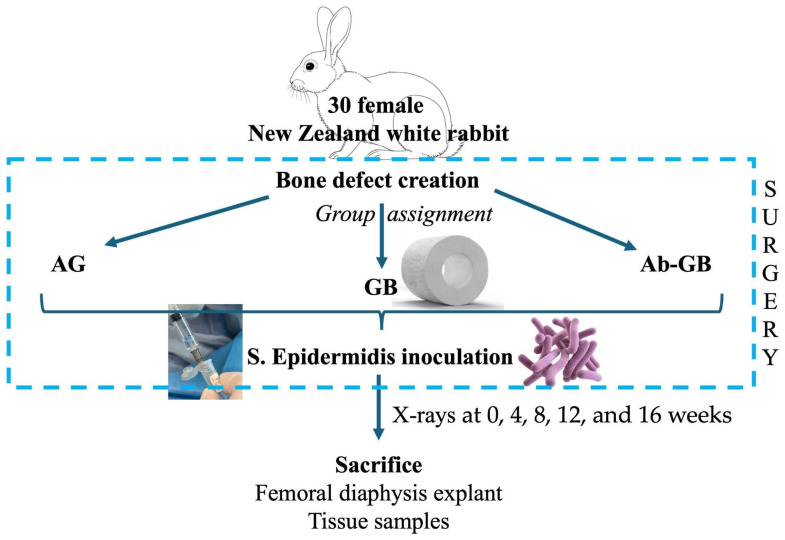
Flow diagram of the study design. (AG: allograft; GB: greenbone; Ab-GB: antibiotic-loaded greenbone).

Thirty skeletally mature New Zealand white rabbits were used. As shown in Figure 1, the rabbits were assigned in a ratio 1:1:1 to one of three experimental groups: (A) antibacterial GreenBone scaffold, (B) non-loaded GreenBone scaffold, and (C) rabbit bone allograft. A critical-size femoral defect was surgically created. At the end of surgery, the bone defect was inoculated percutaneously with 0.5 × 10^7^ colony-forming units (CFU)/0.5 mL of *Staphylococcus epidermidis* (ATCC^®^ 12228™, LGC Standards, Sesto San Giovanni, Italy). Radiographic evaluations were performed at 0, 4, 8, 12, and 16 weeks. Macroscopical, micro-CT, and histological analyses were performed after euthanasia at 16 weeks. Moreover, blood and tissue samples were collected to evaluate systemic and immunological reactions to the implant. 

### 2.2. Ethics Statement

The study was approved by the University of Milan Animal Welfare Organization (n. 17/2019-UT). The animals were housed in the University of Milan Veterinary Hospital (Lodi, Italy). The animals were regularly checked by a certified veterinarian responsible for health monitoring and animal wellbeing supervision. All surgical procedures were performed under general anesthesia, and all efforts were made to minimize suffering.

### 2.3. Animal Model

Thirty female New Zealand white rabbits were included in this study. The animals were provided by Charles River Laboratories Italia s.r.l. (Sant’Angelo Lodigiano, Italy).

### 2.4. Preparation of Bone Substitutes

We used cylindric resorbable biomimetic scaffolds (GreenBone Ortho, Faenza, Italy) with a diameter of 10 mm and a length of 10 mm, weighing approximately 1 g. To prepare the antibiotic-loaded scaffold matching the in vitro tests, the implants were immersed in a gentamicin (40 mg/mL commercially available) and vancomycin (83.3 mg/mL obtained by diluting 500 mg of powder in 6 mL of saline) solution for 2 min before being implanted at the surgical site, according to the manufacturer’s instructions. The femoral segments resected from previously operated rabbits were used as allografts, after lavage with sterile saline and deep-freezing (bone samples were placed in a −80 °C freezer for 30 days).

### 2.5. Surgical Procedures

The rabbits were anesthetized via the inhalation of isoflurane (3%; Merial, Milano, Italy). All animals received a preoperative intramuscular single injection of cefazolin (30 mg/kg; Cefamezin, Teva, Italy). The left lower limb was shaved and disinfected with 2% chlorhexidine gluconate and 70% isopropyl alcohol solution. The animals were placed on sterile drapes; the bodies were covered with sterile sheets to prepare the surgical field. A routine lateral approach to the femur was made. Skin and fascia were incised over 6–7 cm in length. The femoral shaft was exposed by blunt dissection of the muscular tissues. The external fixator-assisted plating osteosynthesis was performed according to a previously published surgical technique, which allows for the mispositioning and dislocation of the plate, and makes surgical procedures fast and standardized [12]. A 10 mm mid-diaphyseal femoral defect was then created using an oscillating saw (Precision Thin 9.0 X 0.38. 25.0 mm, Stryker, Portage, MI, USA). A bone substitute (allograft or scaffold) was inserted into the defect. The femur was stabilized with a Stainless Steel 2.4 mm Locking-Compression Plate (Depuy Synthes Vet, Johnson & Johnson, New Brunswick, NJ, USA), 7 holes, 56 mm in length, and screws. Conventional screws were inserted eccentrically, to further compress the scaffold, if additional stability was needed. The muscular planes were closed with a continuous suture with Vicryl 2/0 and the skin with an intradermic technique (Vicryl Rapid 3/0).

### 2.6. Radiographic Evaluations

Radiographic examinations with X-rays were performed by blinded evaluators at the Lodi University Veterinary Hospital at 0, 4, 8, 12, and 16 weeks following appropriate anesthetic premedication. The first examination was conducted on the same day as the surgery, immediately after the procedure, while the final examination took place on the day of the animal’s sacrifice. 

### 2.7. Sacrifice and Explant

No postoperative therapy was required. Sixteen weeks after the surgery, blood samples were collected, and the animals were subjected to pharmacological euthanasia. The left lower limb was shaved using an electric razor. The animals were positioned on the operating table, and the limb was disinfected with a 2% chlorhexidine gluconate solution and 70% isopropyl alcohol. A sterile disposable drape was used to prepare a surgical field.

Using the same lateral approach to the femur, the fixation devices were removed, and bone consolidation was mechanically assessed. Under sterile conditions, three tissue samples were collected from the bone defect site for microbiological examination. Finally, the left femoral diaphysis was explanted and placed in appropriate containers and immersed in a 4% buffered formaldehyde solution. The specimens were collected and sent to the Pathology Service for macroscopic analysis and histological examination. 

### 2.8. Hematological and Microbiological Examinations

Blood samples collected on the day of sacrifice, prior to euthanasia, were analyzed at the diagnostic pathology laboratory service of the Lodi University Veterinary Hospital for a complete blood test. The tissue samples collected during the explantation procedure were analyzed for microbiological and culture examinations at the same diagnostic pathology laboratory service of the Lodi University Veterinary Hospital. 

### 2.9. Tissue Processing and Slide Preparation

The histological examination of the explanted specimens was conducted at the Pathology Service of the Humanitas Research Hospital. The specimens were processed according to the following steps:

- Fixation in 4% buffered formaldehyde solution for 24 h.
- The orientation of the sample and the marking of the proximal end with India ink.
- The longitudinal cutting of the specimen using a diamond band saw (EXAKT302, EXAKT Advanced Technologies, Norderstedt, Germany).
- Photographing the specimen.
- Additional fixation (post-cutting) in 4% buffered formaldehyde (formalin) solution for 24 h.
- Decalcification in an 8% HCl and 10% CH₂O₂ solution in ddH₂O for no more than 12 h, with overnight formalin before the subsequent cycle. Decalcification was complete when the specimen was rubbery and the tip of the scalpel smoothly penetrated the cortical bone.
- Further fixation (post-decalcification) in formalin solution for 12 h.
- Standard histological preparation:
  ○ Tissue processing.
  ○ Embedding in paraffin using macro-cassettes.
  ○ Cutting into macro-sections with a thickness of 2.5 μm.
  ○ Staining with hematoxylin and eosin.

### 2.10. Histological Examination

The pathologist was blind to the treatment group. Furthermore, to ensure an objective and measurable histological analysis of the samples, two evaluation scales described in the literature were applied. The Histopathological Osteomyelitis Evaluation Score (HOES) [13] enables semiquantitative assessment based on five criteria. Each of these five criteria is assigned a score from 0 to 3 according to a semiquantitative evaluation of the area, as shown in Table 1.

We adopted a second scale to evaluate the histological maturity of newly formed bone [14]. This scale provides a histological grading by measuring the presence of woven bone and lamellar bone and their relative percentages. Compared to the grading system proposed by the authors, we made a minor modification by introducing the possibility of a grade 0 in cases of the absence of new bone formation. We deemed it necessary to apply this scale separately for newly formed bone at the intramedullary level and at the cortical level. Therefore, to describe the maturity of newly formed bone tissue at the intramedullary level, we adapted the scale as detailed in Table 2.

Finally, we distinguished between newly formed bone at the proximal and distal osteotomy sites. This approach led to the evaluation of four quadrants:

- Proximal cortical;
- Proximal intramedullary;
- Distal cortical;
- Distal intramedullary.

The results were recorded in Google Form.

### 2.11. Statistical Analysis 

Data are described as frequencies and percentages for qualitative variables, as means and standard deviations (SDs). Statistical analysis was performed to assess significant differences between the experimental groups. The chi-square (χ^2^) test with the Fisher correction was used to compare the categorical variables across the groups. All statistical tests were performed using a two-sided approach and were considered significant when the *p*-value was inferior to 0.05. All analyses were performed with Stata version 17.

## 3. Results

### 3.1. Mechanical Complications

Eight rabbits were excluded from the study for mechanical complications. In fact, in these eight rabbits, conventional screws were inserted eccentrically in compression mode, to stabilize the plate to the distal fragment and further compress the scaffold in the defect. 

These animals underwent early hardware loosening and seven of them developed non-union (87.5%), a higher percentage compared to the other rabbits (31.8%; *p* = 0.007). Although all of these eight animals (100%) developed osteomyelitis, the difference between them and the other animals (72.7%) was not statistically significant (*p* = 0.099). Indeed, we excluded these eight animals from the final analysis of the results as three belonged to the group with antibiotic-loaded scaffolds, four to the group with non-antibiotic-loaded scaffolds, and one to the allograft group.

The failure was attributed to an unbalanced fixation, characterized by a rigid proximal construct (locking screws) paired with a more flexible distal fixation (conventional screws). It was therefore deemed preferable to sacrifice rabbits 6, 7, 9, 11, and 12 earlier (9 weeks after the surgery). As soon as we detected this complication, we stopped using conventional screws in compression mode and we completed the remaining procedures using only locking screws. Consequently, 22 rabbits were included in the final analysis.

In rabbit 17, a mechanical complication caused by scaffold mobilization occurred. The implant showed signs of instability on the immediate postoperative radiograph. Subsequent radiographs confirmed its complete displacement from the defect. The rabbit belonged to the antibiotic-loaded scaffold group, and the defect did not consolidate.

Moreover, in rabbit 30, an intraoperative fracture occurred at the level of the second proximal screw, which we fixed with a 0 Vicryl wire, achieving good stability. The rabbit belonged to the allograft group, and the defect was consolidated by the end of the experimental period.

Finally, we observed other mechanical complications in rabbits 17 and 22 (both belonging to the scaffold group without antibiotic loading and both not consolidated), where at the time of explant, we noted the deformation of the plate, which appeared bent.

### 3.2. Bone Union

**Figure 2:**
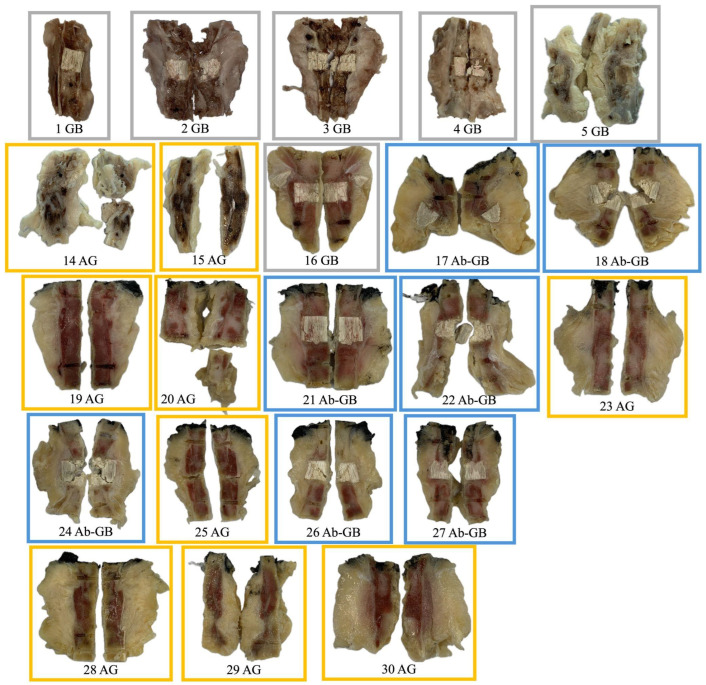
Macroscopic appearance of femoral diaphysis of rabbits included in the analysis (after explantation, fixation, decalcification, and sectioning).

The remaining 22 rabbits are distributed across the three experimental groups as described in Table 3. Their macroscopic aspect is shown in Figure 2.

**Figure 3:**
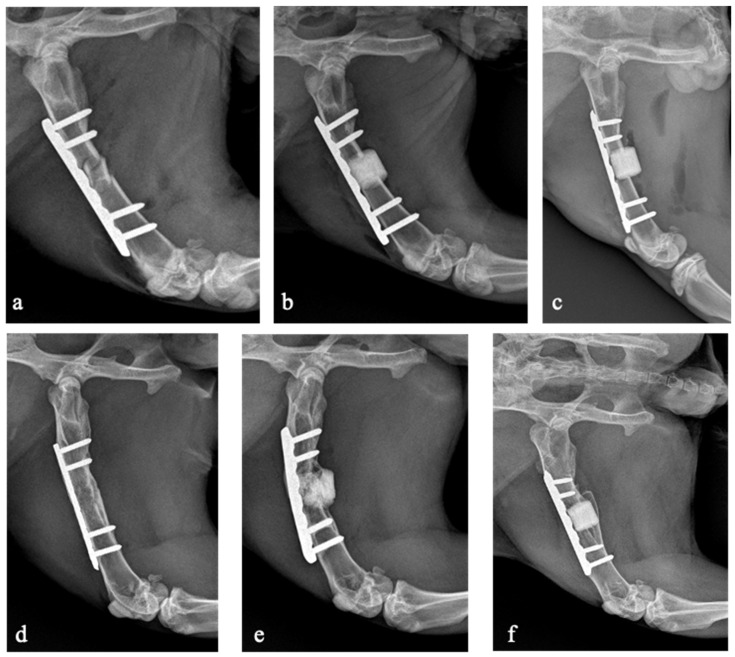
X-rays taken on the day of the surgical procedure (**a**–**c**) and after 16 weeks at the last follow-up (**d**–**f**). Rabbit 23, belonging to the allograft group, developed bone union (**a**,**d**). Rabbit 22, belonging to the antibiotic-loaded GreenBone group, developed non-union (**b**,**e**). Rabbit 1, belonging to the GreenBone group, developed bone union (**c**,**f**).

Seven rabbits (31.8%) developed non-union including five in the antibiotic-loaded group and two in the allograft group. No rabbits developed non-union in the non-loaded scaffold group. The bone union rate was significantly lower in the antibiotic-loaded group (28.6%) compared to the non-antibiotic-loaded groups (86.7%; *p* = 0.006). There were no statistically significant differences in bone union rate between animals treated with the non-loaded scaffold and those treated with the allograft (*p* = 0.215). The X-rays of three rabbits belonging to the three experimental groups are shown in Figure 3.

### 3.3. Microbiological Analysis

The blood tests performed did not reveal significant abnormalities suggestive of systemic inflammatory reactions. The microbiological and culture examination was positive in only two rabbits (4 and 7). Rabbit 7 exhibited clear signs of infection, including a large collection visible on radiographs. During the explant procedure, upon incision, a substantial amount of purulent material was observed. 

### 3.4. Histological Examination for Infection Evaluation

An experienced pathologist reviewed the histological images and completed the HOES osteomyelitis evaluation scale as described above. The resulting distribution across the different categories is shown in Table 3.

In total, 16 rabbits (72.7%) exhibited a histological presentation of osteomyelitis, including eleven cases of chronic osteomyelitis, three cases of silent chronic osteomyelitis, and two cases of active chronic osteomyelitis. Seven of these rabbits belonged to the antibiotic-loaded group, four to the non-loaded scaffold group, and five to the allograft group. No rabbits showed signs of acute osteomyelitis, as expected, given that the explants were performed 16 weeks after bacterial inoculation, reflecting a chronic infection profile. Six rabbits (27.3%) showed no signs of osteomyelitis including two in the non-loaded scaffold group and four in the allograft group. As described in Table 4, in the group of rabbits with antibiotic-loaded implants, we observed a higher rate of osteomyelitis (100%) compared to the rabbits with non-antibiotic-loaded implants (60%; *p* < 0.05). No statistically significant differences were detected in the infection rate between the non-loaded scaffold group and the allograft group (*p* = 0.667). 

**Figure 4:**
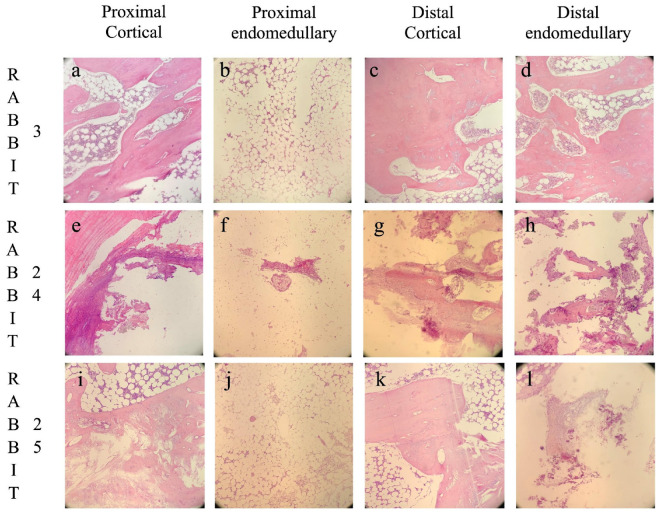
Histological images divided by the four different analyzed areas: proximal cortical (**a**,**e**,**i**), proximal intramedullary (**b**,**f**,**j**), distal cortical (**c**,**g**,**k**), and distal intramedullary (**d**,**h**,**l**). Rabbit 3, belonging to the GreenBone group, developed bone union with no signs of osteomyelitis (**a**–**d**). Rabbit 24, belonging to the antibiotic-loaded GreenBone group, developed non-union with chronic osteomyelitis (**e**–**h**). Rabbit 25, belonging to the allograft group, developed non-union with subsided chronic osteomyelitis (**i**–**l**). Original magnification 100×.

Histological images are shown in Figure 4.

### 3.5. Histological Examination for Bone Maturation Grading

**Figure 5:**
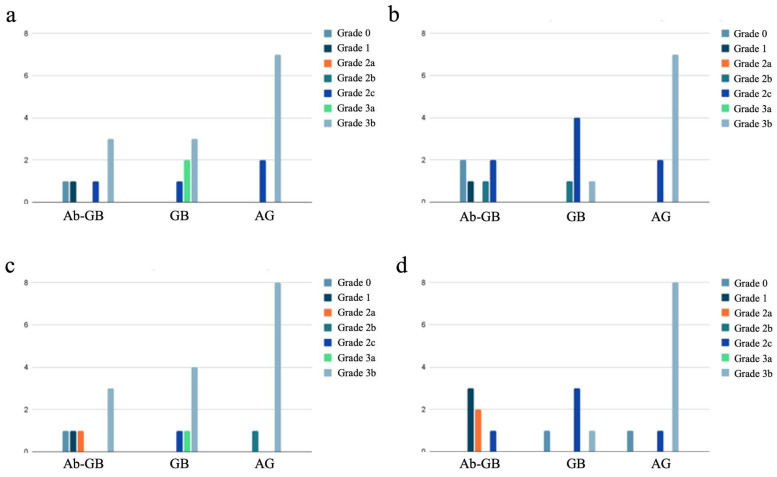
Distribution of newly formed bone maturity grades divided by experimental groups into the four different areas: proximal cortical (**a**), proximal intramedullary (**b**), distal cortical (**c**), and distal intramedullary (**d**).

The distribution of newly formed bone maturity grades divided by experimental groups into the four different areas is shown in Figure 5. 

In the group of rabbits with antibiotic-loaded implants, we observed a lower degree of bone callus maturation (22.2% of HOES grade 3) compared to the rabbits with non-antibiotic-loaded implants (69.5% of HOES grade 3; *p* < 0.001), as shown in Table 4. This difference suggests that the presence of antibiotics in the implant may influence the bone healing process, potentially affecting the progression of callus maturation.

## 4. Discussion

This study investigates the potential of combining antibiotics with biodegradable bone substitutes as a viable treatment option for infected bone defects, utilizing an in vivo rabbit model. Surprisingly, we found that antibiotic-loaded biodegradable bone substitutes may hinder bone healing of segmental bone defects, showing lower union rates and delayed maturation compared to unloaded scaffolds and allografts.

The term “antibiotic” originates from the Greek words “anti”, meaning “against”, and “bios”, meaning “life”, referring to their primary function of inhibiting or destroying bacterial life. This derivation, however, also poses a more general query—can the same characteristics that render medicines successful against bacteria also obstruct the biological mechanisms required for bone regeneration and healing? The study’s surprising findings called into question the widely held notion that using antibiotics in conjunction with bone replacements always promotes infection healing and bone union. 

While a previous in vitro study had demonstrated that antibiotic-loaded scaffolds do not adversely affect mesenchymal stem cell (MSC) viability, colonization, or osteogenic differentiation [15], the current in vivo findings suggest a different outcome. The unexpected higher non-union rate observed in the antibiotic-loaded scaffold group suggests that antibiotics may disrupt the bone healing process. Moreover, the higher osteomyelitis rate in the antibiotic-loaded scaffold group even questions their effectiveness in controlling infection, suggesting a more complex sequence of events leading an osteomyelitis to arise following the repair of a segmental bone defect.

These results have important implications for clinical practice. In fact, antibiotic-loaded bone substitutes are widely used in orthopedic surgery, but the potential trade-offs between infection control and bone regeneration must be carefully considered. Many related studies have primarily focused on periprosthetic joint infection (PJI), and few investigations have addressed the specific problem of fracture-related infections (FRIs) where bone union is the priority [11].

While antibiotic-loaded scaffolds have been extensively studied for their efficacy in managing infections, the current results align with the emerging literature suggesting that high local antibiotic concentrations may have unintended effects on bone regeneration. There is substantial evidence both in vitro and in vivo that antibiotics have several deleterious effects on bone healing and the mechanism of this effect varies depending on the class of antibiotic [16]. Indeed, Beuttel et al. [17] performed a study to investigate the influence of gentamicin-loaded bone graft on the healing of drill hole bone defects in a sheep model. The gentamicin-treated defects showed impaired healing resulting in significantly less bone as shown by µCT and histological analysis. The authors concluded that, when using local gentamicin, a dose-dependent, compromising effect on bone healing should be considered. Furthermore, Freischmidt et al. [18] showed that resorbable calcium sulphate–hydroxyapatite loaded with gentamicin does not have added benefits in infected non-unions in terms of osteoconduction and mechanical bone stability, especially in those with segmental bone defects. The osteoconductive and anti-infective potential could not be demonstrated in the infected situation in this model. Additionally, clinical results published by Ferguson et al. in a case series of 195 long-bone infections, including 110 infected fractures, demonstrated how a biodegradable antibiotic-loaded calcium sulphate bone substitute was an effective antibiotic carrier, despite being associated with poor bone formation [19].

Although their effect on bone healing remains unclear, the use of antibiotic-loaded bone graft substitutes has increased in recent years. A study by Rupp et al. showed that antibiotic-loaded bone graft substitutes were being implanted more frequently with an overall increase in absolute numbers of +194%, rising from 2657 in 2008 to 7811 in 2018. The proportion of bone graft substitutes with added antibiotics has increased too [20]. Nonetheless, this study further confirms that local antibiotics, when added to bone graft substitutes, may hinder the bony healing process; indeed, a lower degree of bone callus maturation (22.2% of HOES grade 3) was found in antibiotic-loaded implant when compared to non-antibiotic-loaded implants (69.5% of HOES grade 3; *p* < 0.001). 

Hence, when employing these materials to address bone defects, clinicians should use caution, especially when bone union is the main objective. Most recommendations on the treatment of bone infections are based on studies conducted on periprosthetic joint infections where infection control is the main target and bone healing is not considered [21]. On the other hand, in infected non-unions and bone defects, bone regeneration is definitely the primary focus; in such scenarios, the detrimental effects on bone repair may be lessened with the use of techniques to improve antibiotic release profiles, eventually avoiding local delivery to preserve the healing potential of the treated bone defect. 

Nonetheless, several limitations of the present study should be noted. First, this study included few animals and the number of animals was further reduced because some that were excluded due to the occurrence of a mechanical complication. Indeed, eight animals were excluded due to mechanical complications resulting from an improper screw fixation technique. After the fixation technique was changed to only locking screws, mechanical complications were drastically reduced in all treatment groups. Nonetheless, the final cohort was reduced to 22 animals. It is worth mentioning that the presence of low frequencies also might have affected chi-square test reliability even though we used the Fisher correction to prevent this risk. Second, the study utilized a rabbit model, which, while widely accepted, may not fully replicate the complexities of human bone healing. Third, the antibiotic release kinetics of the scaffolds were not explicitly quantified, leaving unanswered questions about the local concentrations achieved and their duration. Moreover, a combination of gentamicin (40 mg/mL) and vancomycin (83.3 mg/mL) was used following the manufacturer’s indications and, at present, it is not possible to determine if the detrimental effect on bone healing emerged as a result of this specific combination or was only due to one of the two drugs. Similarly, we tested a single bacterium at a single concentration to mimic the infected bony defect—the inoculation of *S. epidermidis* might not reflect the more complex scenarios often present in clinical practice. Lastly, the study focused on a specific scaffold type loaded with gentamicin and vancomycin, limiting the generalizability of the findings to other materials or drug combinations.

Future studies should focus on elucidating the mechanisms underlying the observed inhibitory effects of antibiotics on bone healing. Additionally, those should test different antibiotics and dosages on similar animal models to determine the effect of each specific combination. Lastly, a significant effort should be undertaken to explore the crucial role of biomechanical stability not only in bone healing but especially in fracture-related infections [22,23].

## 5. Conclusions

The main finding of the present animal study is that antibiotic-loaded biodegradable bone substitutes, used to treat infected bone defects, showed lower union rates and delayed maturation compared to unloaded scaffolds and allografts. Those results further confirm that local antibiotics may hinder the bony healing process and that future research should focus on developing innovative and effective strategies to address infection without impeding the bone healing processes. 

## Figures and Tables

**Table 1:** Histopathological Osteomyelitis Evaluation Score (HOES) template.

	Non-existent = 0	Mild = 1	Moderate = 2	Severe = 3
evaluation in a three-part step		⅓ of the section area	⅔ of the section area	the entire section area
A1: Osteonecrosis				
A2: Soft tissue necrosis				
A3: Granulocytic infiltration				
**Sum of A1 to A3**	≥ 4 → signs of acute osteomyelitis;			
C1: Bone neoformation/fibrosis				
C2: Lymphocytic–macrophagic infiltration				
**Sum of C1 to C2**	≤1 → no signs of osteomyelitis; ≤4 → signs of subsided (calmed) osteomyelitis; ≥4 → signs of chronic osteomyelitis;			
**Sum of A1 to A3 and C1 to C2**	≥6 signs of active chronic osteomyelitis.			

The table is not intended to present experimental results, but rather to illustrate the data collection framework applied during histological analysis. Therefore, numerical values and specific entries are not included, as the table has been filled out on a case-by-case basis during the evaluation process.

**Table 2:** Bone maturation grading system template.

Cortical	Intramedullary
0. Absence of newly formed bone.*	
1. Presence of discontinuous spicules of woven bone.	
2. Presence of both woven bone and lamellar bone:	
2a. Woven bone predominant over lamellar bone;	
2b. Woven bone and lamellar bone present in equal proportions;	
2c. Lamellar bone predominant over woven bone.	
3. Presence of lamellar bone only:	
3a. Partially compact Haversian systems with significant intracortical space between osteons;	3a. Trabecular bone; *
3b. Fully compact mature osteons with lamellar bone between osteons and little to no intracortical space.	3b. Trabecular bone with islands of bone marrow. *

* authors’ modifications of the grading system.

**Table 3:** Results in terms of bone union, infection, and callus maturation divided by experimental groups.

		Ab-GB (*n* = 7)	GB (*n* = 6)	AG (*n* = 9)
**Bone union**	**Non-union**	5	0	2
	**Union**	2	6	7
**Infection**	**Chronic OM**	6	1	4
	**Subsided OM**	1	1	1
	**Chronically florid OM**	0	2	0
	**No signs of OM**	0	2	4
Bone maturation *	**0**	7	1	1
	**1**	6	0	0
	**2a**	3	0	0
	**2b**	1	1	1
	**2c**	4	9	5
	**3a**	0	3	0
	**3b**	6	9	29
	**N/A**	1	1	0

* *n* > 22 because every sample was analyzed in four different quadrants: proximal cortical area, proximal intramedullary area, distal cortical area, and distal intramedullary area. OM: osteomyelitis; AbGB: antibiotic-loaded GreenBone scaffold; GB: GreenBone scaffold; AG: allograft.

**Table 4:** Results in terms of bone union, infection, and callus maturation in antibiotic-loaded scaffolds compared to non-antibiotic-loaded bone substitutes.

		Ab-GB (*n* = 7)	GB+AG (*n* = 15)
**Bone union** *p* = 0.006	**Non-union, *n* = 7 (31.8%)**	5 (71.4%)	2 (13.3%)
	**Union, *n* = 15 (68.2%)**	2 (28.6%)	13 (86.7%)
**Infection** *p* < 0.05	**Chronic OM—*n* = 16 (72.7%)**	7 (100%)	9 (60%)
	**No signs of OM—*n* = 6 (27.3%)**	0	6 (40%)
Bone maturation * p < 0.001	**0–1**	13	2
	**2**	8	16
	**3**	6	41

* *n* > 22 because every sample was analyzed in four different quadrants: proximal cortical area, proximal intramedullary area, distal cortical area, and distal intramedullary area. OM: osteomyelitis; AbGB: antibiotic-loaded GreenBone scaffold; GB: GreenBone scaffold; AG: allograft.

## Data Availability

The data that support the findings of this study are available from the corresponding author F.V. upon reasonable request.

## Abbreviations

The following abbreviations are used in this manuscript:

OM	Osteomyelitis
AbGB	Antibiotic-loaded GreenBone
GB	GreenBone
AG	Allograft
SBDs	Segmental bone defects
CFU	Colony forming unit
HOES	Histopathological Osteomyelitis Evaluation Score

## References

[1] Mauffrey C., Barlow B.T., Smith W. (2015). Management of Segmental Bone Defects. J. Am. Acad. Orthop. Surg..

[2] Giannoudis P.V., Dinopoulos H., Tsiridis E. (2005). Bone substitutes: An update. Injury.

[3] Campana V., Milano G., Pagano E., Barba M., Cicione C., Salonna G., Lattanzi W., Logroscino G. (2014). Bone substitutes in orthopaedic surgery: From basic science to clinical practice. J. Mater. Sci. Mater. Med..

[4] Nauth A., McKee M.D., Einhorn T.A., Watson J.T., Li R., Schemitsch E.H. (2011). Managing Bone Defects. J. Orthop. Trauma.

[5] Nauth A., Crist B.D., Morshed S., Watson J.T., Pape H.-C. (2023). Management of aseptic nonunions and severe bone defects: Let us get this thing healed!. OTA Int. Open Access J. Orthop. Trauma.

[6] Calori G., Mazza E., Colombo M., Ripamonti C. (2011). The use of bone-graft substitutes in large bone defects: Any specific needs?. Injury.

[7] Yang Y.P., Labus K.M., Gadomski B.C., Bruyas A., Easley J., Nelson B., Palmer R.H., McGilvray K., Regan D., Puttlitz C.M. (2021). Osteoinductive 3D printed scaffold healed 5 cm segmental bone defects in the ovine metatarsus. Sci. Rep..

[8] Henkel J., Savi F.M., Berner A., Fountain S., Saifzadeh S., Steck R., Epari D.R., Woodruff M.A., Knackstedt M., Schuetz M.A. (2021). Scaffold-guided bone regeneration in large volume tibial segmental defects. Bone.

[9] Filardo G., Kon E., Tampieri A., Cabezas-Rodríguez R., Di Martino A., Fini M., Giavaresi G., Lelli M., Martínez-Fernández J., Martini L. (2013). New Bio-ceramization process applied to vegetable hierarchical structures for bone regeneration: An experimental model in sheep. Tissue Eng. Part A.

[10] Minardi S., Corradetti B., Taraballi F., Sandri M., Van Eps J., Cabrera F.J., Weiner B.K., Tampieri A., Tasciotti E. (2015). Evaluation of the osteoinductive potential of a bio-inspired scaffold mimicking the osteogenic niche for bone augmentation. Biomaterials.

[11] Metsemakers W.-J., Fragomen A.T., Moriarty T.F., Morgenstern M., Egol K.A., Zalavras C., Obremskey W.T., Raschke M., McNally M.A., Fracture-Related Infection (FRI) consensus group (2020). Evidence-Based Recommendations for Local Antimicrobial Strategies and Dead Space Management in Fracture-Related Infection. J. Orthop. Trauma.

[12] Vandenbulcke F., Anzillotti G., Ravasio G., Malagoli E., Conte P., Balzarini B., Kirienko A., Kon E. (2023). External fixator-assisted plating osteosynthesis in a rabbit model of femoral bone defects appears to be a feasible and reproducible surgical technique: Preliminary insights from a bone substitute study. J. Exp. Orthop..

[13] Tiemann A., Hofmann G.O., Krukemeyer M.G., Krenn V., Langwald S. (2014). Histopathological Osteomyelitis Evaluation Score (HOES)—An Innovative Approach to Histopathological Diagnostics and Scoring of Osteomyelitis. GMS Interdiscip. Plast. Reconstr. Surg. DGPW.

[14] Shapiro F., Maguire K., Swami S., Zhu H., Flynn E., Wang J., Wu J.Y. (2021). Histopathology of osteogenesis imperfecta bone. Supramolecular assessment of cells and matrices in the context of woven and lamellar bone formation using light, polarization and ultrastructural microscopy. Bone Rep..

[15] Salamanna F., De Luca A., Vandenbulcke F., Di Matteo B., Kon E., Grassi A., Ballardini A., Morozzi G., Raimondi L., Bellavia D. (2024). Preliminary osteogenic and antibacterial investigations of wood derived antibiotic-loaded bone substitute for the treatment of infected bone defects. Front. Bioeng. Biotechnol..

[16] Kallala R., Graham S.M., Nikkhah D., Kyrkos M., Heliotis M., Mantalaris A., Tsiridis E. (2011). *In vitro* and *in vivo* effects of antibiotics on bone cell metabolism and fracture healing. Expert Opin. Drug Saf..

[17] Beuttel E., Bormann N., Pobloth A.-M., Duda G.N., Wildemann B. (2019). Impact of Gentamicin-Loaded Bone Graft on Defect Healing in a Sheep Model. Materials.

[18] Freischmidt H., Armbruster J., Rothhaas C., Titze N., Guehring T., Nurjadi D., Kretzer J.P., Schmidmaier G., Grützner P.A., Helbig L. (2022). Efficacy of an Antibiotic Loaded Ceramic-Based Bone Graft Substitute for the Treatment of Infected Non-Unions. Biomedicines.

[19] Ferguson J.Y., Dudareva M., Riley N.D., Stubbs D., Atkins B.L., McNally M.A. (2014). The use of a biodegradable antibiotic-loaded calcium sulphate carrier containing tobramycin for the treatment of chronic osteomyelitis: A series of 195 cases. Bone Jt. J..

[20] Rupp M., Klute L., Baertl S., Walter N., Mannala G., Frank L., Pfeifer C., Alt V., Kerschbaum M. (2021). The clinical use of bone graft substitutes in orthopedic surgery in Germany—A 10-years survey from 2008 to 2018 of 1,090,167 surgical interventions. J. Biomed. Mater. Res. Part B Appl. Biomater..

[21] Metsemakers W., Morgenstern M., McNally M., Moriarty T., McFadyen I., Scarborough M., Athanasou N., Ochsner P., Kuehl R., Raschke M. (2018). Fracture-related infection: A consensus on definition from an international expert group. Injury.

[22] Santolini E., Giordano V., Giannoudis P.V. (2024). Effect of mechanical stability of osteosynthesis on infection rates: Timing of temporary and definitive fixation. Injury.

[23] Foster A.L., Moriarty T.F., Zalavras C., Morgenstern M., Jaiprakash A., Crawford R., Burch M.-A., Boot W., Tetsworth K., Miclau T. (2021). The influence of biomechanical stability on bone healing and fracture-related infection: The legacy of Stephan Perren. Injury.

